# Adapting Social Media Recruitment Strategies to the Preferences of Spanish-Speaking Older Adults

**DOI:** 10.1093/geroni/igaf122.585

**Published:** 2025-12-31

**Authors:** Michelle Villar, Monica Rosselli, Jinwoo Jang, Borko Furht, Ruth Tappen

**Affiliations:** Florida Atlantic University, Lake Worth, Florida, United States; Florida Atlantic University, Boca Raton, Florida, United States; Florida Atlantic University, Boca Raton, Florida, United States; Florida Atlantic University, Boca Raton, Florida, United States; Florida Atlantic University, Boca Raton, Florida, United States

## Abstract

Social media is proving to be a game changer in the recruitment of older adults for research. Given the success of our initial Facebook outreach to older adults in South Florida, we expanded it to include older Spanish-speaking adults. Neither of these initial campaigns yielded Spanish-speaking participants. In our second campaign thus far, follow-up telephone outreach yielded successful contact with 11 of 39 (28%) leads. Of those 11, 2 were excluded due to distance from the testing sites, 3 did not want further information, and 6 declined after receiving more information. Many reported difficulty fully understanding the content and intent of the posted Facebook ads. Our more traditional recruitment efforts utilized personal referrals and health fairs to foster a critical personal connection, which appeared to be lacking on the social media platform. This disconnect highlighted the need to provide a more complete description of the participant’s role in the study and achieve relatability with the target audience in the ads. We concluded that translation into Spanish was insufficient to engage potential participants, and an additional step is necessary, i.e., incorporating personal experiences of Spanish-speaking participants already enrolled in the study and a voiceover that clearly articulates the study’s purpose, benefits, and requirements. By fostering meaningful connections and providing appropriate messaging, researchers can attract individuals genuinely interested in participation. Ultimately, adapting recruitment strategies to reflect participants’ values and preferences can increase the likelihood of successful engagement and improve the effectiveness of participant recruitment efforts.

